# Skin‐Interfaced Bifluidic Paper‐Based Device for Quantitative Sweat Analysis

**DOI:** 10.1002/advs.202306023

**Published:** 2023-12-22

**Authors:** Muhan Deng, Xiaofeng Li, Kui Song, Hanlin Yang, Wenkui Wei, Xiaojun Duan, Xiaoping Ouyang, Huanyu Cheng, Xiufeng Wang

**Affiliations:** ^1^ School of Materials Science and Engineering Xiangtan University Xiangtan Hunan 411105 China; ^2^ Department of Engineering Science and Mechanics Xiangtan University Xiangtan Hunan 411105 China; ^3^ Hunan Provincial Children's Hospital Changsha Hunan 410000 China; ^4^ Department of Engineering Science and Mechanics The Pennsylvania State University University Park PA 16802 USA

**Keywords:** distance‐based metric approach, paper‐based microfluidic device, sweat chloride, sweat glucose, sweat rate and volume

## Abstract

The erratic, intermittent, and unpredictable nature of sweat production, resulting from physiological or psychological fluctuations, poses intricacies to consistently and accurately sample and evaluate sweat biomarkers. Skin‐interfaced microfluidic devices that rely on colorimetric mechanisms for semi‐quantitative detection are particularly susceptible to these inaccuracies due to variations in sweat secretion rate or instantaneous volume. This work introduces a skin‐interfaced colorimetric bifluidic sweat device with two synchronous channels to quantify sweat rate and biomarkers in real‐time, even during uncertain sweat activities. In the proposed bifluidic‐distance metric approach, with one channel to measure sweat rate and quantify collected sweat volume, the other channel can provide an accurate analysis of the biomarkers based on the collected sweat volume. The closed channel design also reduces evaporation and resists contamination from the external environment. The feasibility of the device is highlighted in a proof‐of‐the‐concept demonstration to analyze sweat chloride for evaluating hydration status and sweat glucose for assessing glucose levels. The low‐cost yet highly accurate device provides opportunities for clinical sweat analysis and disease screening in remote and low‐resource settings. The developed device platform can be facilely adapted for the other biomarkers when corresponding colorimetric reagents are exploited.

## Graphical abstract

Here, a bifluidic‐distance metric approach, with one channel measuring sweat rate and volume, and the other channel analyzing biomarkers based on the collected sweat volume, is presented. This device is suitable for assessing hydration status and monitoring glucose levels. The low‐cost yet highly accurate device provides opportunities for clinical sweat analysis and disease screening in remote and low‐resource settings.

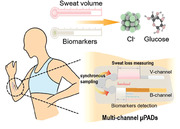

## 1 Introduction

As a potential alternative or auxiliary to blood, sweat provides a non‐invasive source to detect biomarkers for revealing various physiological or psychological processes,^[^ ^1^, ^2^, ^3^ ^]^ leading to applications in sports performance evaluation^[^ ^4^, ^5^, ^6^ ^]^ and disease diagnosis.^[^ ^7^, ^8^ ^]^ For instance, the chloride concentration ([Cl^−^]) in sweat could reveal deficits in bodily fluids and electrolytes caused by exercise or heat stress‐induced sweating,^[^ ^9^ ^]^ which helps assess hydration status and reflect adverse impacts on physical and cognitive conditions. Sweat chloride concentration is also considered the gold standard for quantitatively assessing cystic fibrosis, a common genetic disorder that significantly reduces life expectancy.^[^ ^10^ ^]^ Early diagnosis through sweat chloride assessment is crucial to initiate timely and effective care. Additionally, the correlation between sweat and blood glucose levels promises the use of sweat glucose detection for managing diabetes.^[^ ^11^, ^12^, ^13^ ^]^ Despite the significant interest and manifold benefits of sweat biomarker analysis, the challenge of precisely and rapidly detecting them hampers its use as a clinical standard.

Compared to conventional sweat analysis that involves controlled laboratory settings with coiled tubing and absorbent pads, wearable electronic platforms with electrochemical sensors and wireless communication allow continuous monitoring of various chemical species in sweat.^[^ ^14^, ^15^, ^16^, ^17^ ^]^ However, the complicated calibration procedures and high power consumption present practical challenges for long‐term applications. In comparison, the skin‐interfaced colorimetric microfluidics without any electrical components provides rapid semi‐quantification of both sweat volume and biomarker concentration.^[^ ^18^, ^19^ ^]^ However, continual sweat flow can cause color leaching and non‐uniform color responses. Spatially uneven lighting conditions also affect the accuracy of color analysis. Besides analyte concentration, the collected sweat sample volume also affects the intensity of the colorimetric signal to cause imprecise quantification when the sweat rate information is absent.^[^ ^20^, ^21^ ^]^ Therefore, accurate detection of sweat biomarkers can only be made with the simultaneously measured sweat secretion rate (or sweat volume) and colorimetric signals. Hence, it is imperative to develop advanced quantitative sweat analysis techniques to address these challenges for high‐accuracy sweat analysis.

Although sweat is readily accessible on almost all bodily surfaces, its production can be intermittent, uncertain, and even unpredictable due to changes in physiological or psychological processes.^[^ ^22^, ^23^ ^]^ Besides the challenge in consistent sweat sampling, several other factors such as sweat mixing from different time periods, uneven transport of sweat across sensing sites, and sweat evaporation, may also undermine the accuracy of sweat analysis.^[^ ^24^, ^25^ ^]^ This is particularly problematic for colorimetric sweat sensors, as the transport/reaction rates of analytes and the activity of the reagent rely heavily on the sweat flow rate or instant sampling volume.^[^ ^26^ ^]^ Efforts to address these challenges have led to the exploration of diverse analytical techniques such as volume sensing,^[^ ^21^ ^]^ color overlay,^[^ ^27^, ^28^ ^]^ surface‐enhanced Raman spectrometry,^[^ ^29^ ^]^ and fluorometric modalities^[^ ^30^ ^]^ to quantify sweat electrolytes and metabolites. However, they are not suitable for low‐cost, large‐scale, point‐of‐care applications. One promising solution is to use distance‐based colorimetric detection for visual quantification, similar to reading a thermometer.^[^ ^31^, ^32^ ^]^ The direct readout of the signals does not require any external optical detectors and is not impacted by lighting conditions or surface contamination. Nonetheless, it is challenging to apply it due to fluctuations in the volume of instant sweat secretion during continuous sweating.

Herein, this work reports a skin‐interfaced colorimetric bifluidic sweat device for real‐time accurate quantification of sweat loss and biomarker analysis, even during intermittent sweating. Consisting of two channels, the paper‐based bifluidic device explores one for sweat sampling and quantification and the other one for accurate biomarker analysis based on the calculated collected sweat volume. The device with a closed microfluidic design can also reduce evaporation and resist contamination from the environment for enhanced accuracy. In the proof‐of‐the‐concept demonstration to detect chloride and glucose, the device based on the bifluidic‐distance metric approach provides sweat analysis consistent with laboratory‐based methods, aiding the evaluation of hydration status and glycemic monitoring. The sensing platform can also be extended to detect the other biomarkers when the reagents are replaced.

## 2 Results

### 2.1 Design of the Skin‐Interfaced Bifluidic Sweat Device and Its Sensing Strategies

**Figure 1:**
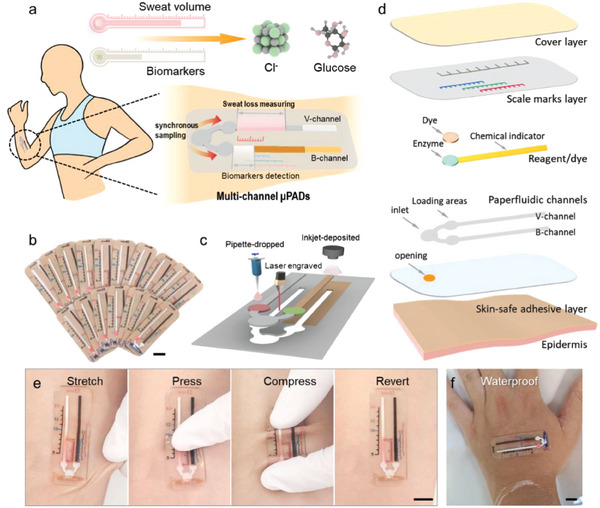
Design of the skin‐interfaced bifluidic sweat device and its sensing strategies. a) Conceptual illustration of a skin‐interfaced bifluidic sweat device based on a bi‐distance metric approach to measure sweat volume and biomarkers from self‐inhalation. b) Photograph of bifluidic sweat devices produced from the same batch. c) Schematic showing the device fabrication process. d) Exploded view of the device to highlight various functional layers. Optical images of the paper‐based fluidic device on the skin e) under various mechanical deformations: stretching, pressing, and compressing, and f) underwater. Scale bar: 1 cm.

A skin‐interfaced bifluidic device introduced here explores a bi‐distance metric approach to collect sweat for volumetric measurement and biomarker analysis after self‐inhalation (**Figure** **1**a). Compared with reported colorimetric sensors, this device eliminates the need to predetermine the sweat volume for real‐time quantitative analysis of biomarker concentrations over varied sweat secretion rates. It also removes the constraints imposed by external devices (e.g., colorimetric cards, smartphone‐based imaging software, and associated concerns over lighting conditions). Additionally, the device features a single inlet to minimize external contamination and sample evaporation. Consequently, it could be used in extreme scenarios, such as aquatic or arid environments, without being affected by back pressure. The cost‐effective and scalable fabrication process (Figure 1b) relies on the combination of laser engraving, inkjet deposition, pipette dropping, and stencil lamination (Figure 1c; Figure S1, Supporting Information), which can be further automated. Based on the requirements for flexibility and sampling volume, the device can be easily customized, tailoring to different users (infants or adults) and various body locations.

The bifluidic device exploits multilayered stacks of thin‐film polymers and filter papers, including a skin‐safe adhesive layer, paper‐based microfluidic channels, colorimetric assay reagent/dye, scale marks layer, and cover layer (Figure 1d). The laser‐machined paper‐based microfluidic channels consisting of different functional filter papers feature a V‐channel with dyes pipetted to measure sweat volume and a B‐channel with inkjet‐deposited chemical indicators and enzymes pipetted to quantitatively measure biomarkers. With a common inlet, synchronous sweat sampling is accomplished. The layer with precisely printed scale marks on a transparent polyester film for direct marker readout is covered by an additional polyester cover layer to protect the scales from water and environmental pollutants. The skin adhesive layer with an opening at the inlet allows device attachment on the skin.

Driven by the sweat pressure and capillary effects, sweat flows to the V‐channel and interacts with the dye to generate information on the sweat volume and rate. Simultaneously, biomarkers in sweat react with reagents deposited on the B‐channel to form a color band. As the sweat continues to flow along the B‐channel, the sweat biomarkers are completely consumed to form a color change that is no longer changing. The biomarker concentration is then obtained as the ratio of the biomarker amount read out from the B‐channel to the sweat volume read out from the V‐channel. The thin device (≈500 µm) with a low bending stiffness provides an improved level of comfort and high device integrity even under various mechanical deformations such as stretching, pressing, and compressing (Figure 1e; Figure S2, Supporting Information). Additionally, the closed design of the microchannels can effectively prevent water infiltration, to allow device operation in aquatic environments (Figure 1f).

### 2.2 Flow Characteristic of the Bifluidic Sweat Device

**Figure 2:**
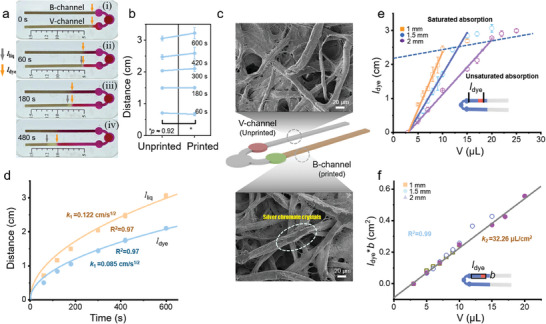
Flow characteristics of the skin‐interfaced bifluidic sweat device. a) Photographs of a device with a 1.5 mm‐wide channel showing the traveling liquid front over time. b) Comparison of water traveling distance over time between unprinted (V‐channel) and printed (B‐channel) filter papers. c) SEM images of the V‐ and B‐channels. d) Relationship between the traveling distance and time for dye and water in the V‐channel. e) Dye traveling distance as a function of the collected liquid volume, and f) the normalized linear relationship.

It is vital to have identical flow characteristics in the V‐channel and B‐channel so the sweat rate/volume obtained from one can be used for biomarker concentration analysis in the other one. The impact of inkjet‐printed precipitate on the flow characteristics is evaluated by comparing the water flow in pre‐ and post‐printed channels (i.e., V‐channel with pristine filter paper versus B‐channel with the inkjet‐printed colorimetric reagent of Ag_2_CrO_4_ for chloride detection). The comparison in water traveling distance over 6 min between the unprinted and printed microfluidic channels (length *l* × width *b* × thickness *δ* of 35 × 1.5 × 0.3 mm) shows a synchronous advancing front (**Figure** **2**a,b). The negligibly small impact from the inkjet deposited Ag_2_CrO_4_ on the flow characteristics is revealed by the SEM (Figure 2c). The microstructural differences between the two indicate that the colorimetric reagents from inkjet deposition are uniformly distributed over the top side of paper fibers without obstructing their voids. In contrast, the precipitate prepared by the conventional pipette‐dropping method forms a composite structure with paper fibers to affect the fluid flow (Figure S3, Supporting Information).

The flow of the dye interacting with sweat in the V‐channel depends on the amount and rate of sweating. The advancing front of the dye (*l*_dye_) progresses through the channel concurrently with the advancing front of liquid (*l*_liq_), but with a consistent lag (Figure 2a), due to the adsorption and retention of dye molecules in voids of the filter paper.^[^ ^33^, ^34^ ^]^ The retardation factor (*R*_f_) can be used to correlate *l*_dye_ and *l*_liq_ for a given device, with the kinetic curves described by the Lucas–Washburn equation^[^ ^35^ ^]^ (Figure 2d). The kinetic behavior of the dye‐wicking front as a function of time *t* can be expressed as:

(1) $$l_{\mathrm{dye}}=\sqrt{\frac{\gamma rR_{f}^{2}\cos\theta }{2\mu }t}\;=k_{1}\;\sqrt{t}$$

where *γ* is the surface tension of the liquid, *r* is the average pore radius, *µ* is the viscosity of the liquid, and *θ* is the contact angle between the liquid and the boundary wall. The combined constant *k*_1_ can be determined by fitting the experimental data.

The measured *l*_dye_ further exhibits a linear dependence on the sweat loss volume for the microfluidic channels with different widths (1, 1.5, and 2 mm) and a length of 35 mm (Figure 2e), before saturation when *l*_liq_ reaches the end of the channel. As the channel width decreases, a larger slope and a higher sensitivity are observed, but narrow channels have reduced water capacity. Considering the balance in the sensitivity and target sweat volume, the width of 1.5 mm is chosen in the subsequent experiments unless otherwise specified. Therefore, the linear relationship between *l*_dye_ and net volume of the collected liquid (*V*, excluding loading areas and inlet volume) follows:

(2) $$V\;=\frac{\delta \rho }{R_{f}}\;b\;l_{\mathrm{dye}}=k_{2}\;bl_{\mathrm{dye}}$$

where *ρ* is the porosity of the filter paper. The constant *k_2_* of 32.26 µL cm^−2^ obtained from the fitted experimental linear curve establishes the relationship between *bl*_dye_ and *V* (Figure 2f), which can be used to directly visualize the collected sweat volume in the V‐channel for a given *b* based on the corresponding predetermine scale (e.g., 10 µL in Figure 2a).

### 2.3 The Bifluidic‐Distance Metric Approach for Quantitative Analysis of Biomarkers

**Figure 3:**
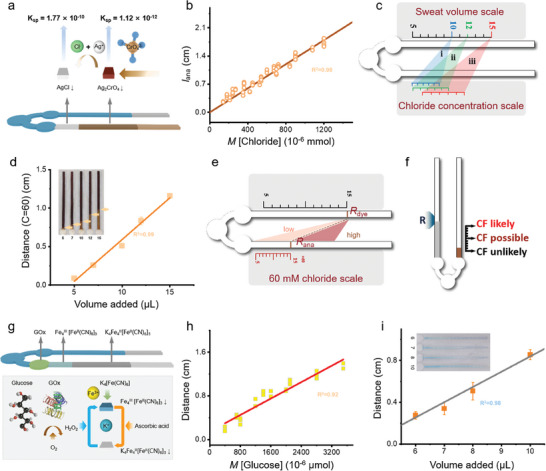
The bifluidic‐distance metric approach for quantitative biomarker analysis. a) Schematic showing the detection of the chloride concentration in paper‐based microfluidic channels. b) Relationship between the color change distance and chloride amount. c) Schematic for the calibrated scale: i) 10 µL, ii) 12 µL, and iii) 15 µL. d) Relationship between the color change distance and the collected volume of artificial sweat with 60 mm chloride. e) Schematic showing the calibrated scale for 60 mm chloride and f) CF. g) Schematic showing the detection of glucose concentration in paper‐based microfluidic channels. Relationship between the color changing distance and h) glucose amount and i) the volume of artificial sweat with 200 µm glucose.

After determining the sweat volume from the V‐channel, the sweat biomarker concentrations can then be quantified with the measurements from the B‐channel. As a representative example, chloride is detected through argentometric determination, a variant of Mohr titration (**Figure** **3**a). The brown silver chromate (Ag_2_CrO_4_) crystals with silver solution deposited on top in the B‐channel react with the chloride solution to change color from brown to white, resulting from the formation of silver chloride precipitate with a lower solubility (1.33 × 10^−5^ mol L^−1^) compared to silver chromate (6.50 × 10^−5^ mol L^−1^). Combining the precipitated distance or the color‐changing length (*l*_ana_) in the B‐channel with the amount of sweat from the V‐channel (*V*) yields the concentration of chloride.

The calibration of the chloride analysis can be achieved by loading standard chloride solutions with varying concentrations and volumes into the device. The solution just in the inlet alongside two circular loading sites may lead to inconspicuous color alteration boundaries within the B‐channel, when a volume of 10 µL or below with a concentration of under 30 mm is used (Figure S4, Supporting Information). Consequently, a solution volume of 10–15 µL (to avoid exceeding the saturation limit of 15 µL) is used for chloride calibration, with 10 µL for pre‐soaking and 0–5 µL for detection in the B‐channel. A clear linear relationship between the amount of chloride *M* (the product of the chloride concentration *C* and total added solution volume) and the color‐changing distance *l*_ana_ (Figure 3b): *M*  = *k*_3_ *l*_ana_, where *k*_3_ is 0.668 × 10^−3^ mmol cm^−1^. Considering the measured sweat volume in Equation (2), the chloride concentration is given as

(3) $$C\;=l_{\mathrm{ana}}k_{3}/V\;$$

For a given collected sweat volume (namely 10, 12, or 15 µL), the calibrated scales in the B‐channel across a range of concentrations from 20 to 120 mm can be obtained (Figure 3c). This bifluidic‐distance metric design offers a convenient means of determining the biomarker concentrations in unknown sweat samples at pre‐set collecting volumes, with the estimates for the other sweat volumes made through interpolation. Attaching the device to the upper back of a volunteer engaged in exercise for three different durations (i.e., 20, 23, and 29 min) produces sweat of the three pre‐set volumes (i.e., 10, 12, or 15 µL). The measured chloride concentration is consistently in the range of 30–40 mm L^−1^ for all measurements on the same human subject (Figure S5, Supporting Information).

The quantitative analysis of the sweat chloride concentration can also help establish the classification of the likelihood of cystic fibrosis (CF): CF unlikely, CF possible, and CF likely, based on the comparison between the measured and the threshold concentration of 60 mm.^[^ ^10^ ^]^ The process starts with the fitting of Equation (3) with the sweat volume reading (*V*_c_) and the chloride assay reading (*L*_ava_) for five different volumes of solutions (5, 7, 10, 12, and 15 µL) with 60 mm chloride (Figure 3d). Diagnosis of the CF can be accomplished if *L*_ava_ exceeds *V*_c_ on the established scale (Figure 3e) according to the linear fitting results. Printing multiple distinguishable points along specified channels allows a simple readout to check when sweat flows near the R point of the V‐channel and the color change in the B‐channel occurs near the three pre‐set feature points (Figure 3f).

Besides chloride, other sweat biomarkers such as glucose and ascorbic acid^[^ ^36^ ^]^ can also be detected. For instance, glucose detection is achieved using the Prussian white (PW)/Prussian blue (PB) system. The sequential inkjet deposition of K_4_[Fe(CN)_6_] and FeCl_3_ onto the B‐channel yields a stable blue complex PB (Fe_4_[Fe(CN)_6_]_3_), followed by depositing excessive ascorbic acid to reduce the PB to PW. The decomposition of glucose into gluconic acid and hydrogen peroxide, catalyzed by glucose oxidase (GOx), transforms PW to PB to result in a detectable color change from white to blue (Figure 3g). Channels with a width of 1 mm are used for increased sensitivity to detect low concentrations of sweat glucose in the detection from 100 to 500 µm, encompassing those for both hyperglycemic patients and healthy individuals. It is worth noting that sweat glucose correlates with blood glucose levels, with a threshold of 200 µm to signify hyperglycemia.^[^ ^37^, ^38^, ^39^ ^]^ With the calibration and adjustment of the scales, the sweat glucose from the human subject can be determined (Figure 3h,i).

### 2.4 Robust Performance for Discontinuous Sweating and Durability Test

**Figure 4:**
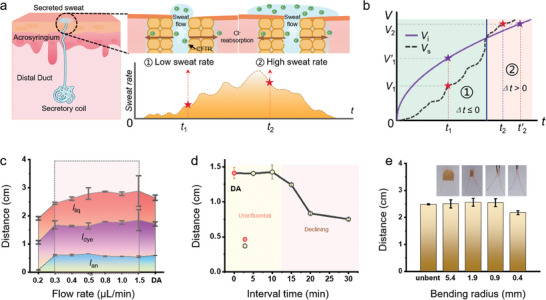
Robust performance for discontinuous sweating and durability test. a) Sweat secretion mechanism of the eccrine sweat gland. b) Sweat secretion rate (*V_s_*) and absorption rate (*V_i_*) in the paper‐based microfluidic channel during exercise. c) Comparison of the reactive precipitated color, dye, and liquid traveling distances at different flow rates. d) Changes in precipitated distances from 5 to 30 min after dropwise adding three droplets of chloride solution to the device. e) Comparison of the distance of the dye traveling in front of the device before and after bending to a radius of 5.4, 1.9, 0.9, and 0.4 mm with added water of 15 µL.

The unpredictable and dynamic nature of human sweating, complicated by the imbibition kinetics of sweat by the porous paper fibers (Note S1, Supporting Information), necessitates the study of the impact of sweat flow rate on measurement accuracy. Regulated by the hypothalamus, sweat rate changes as the number of active glands and expulsion frequency are modulated by nerve impulses.^[^ ^40^, ^41^ ^]^ As physical activity or thermal stress increases, glandular activation is heightened to increase sweat rate (**Figure** **4**a). During the initial exercise, low sweat secretion rates and high absorbency of the paper‐based channel allow for immediate absorption (Region 1 in Figure 4b). However, when the sweat secretion rate increases to exceed the sweat flow rate through the device would lead to unabsorbed sweat and lag time for sweat sampling (Region 2 in Figure 4b).

With an inlet opening of a 2.5 mm radius in the device, sweat can be rapidly collected before saturation within ≈20 min when the sweat rate approaches 2 µL min^−1^ cm^−2^. The negative lag time (*dt*<0) calculated from Equation S6b (Supporting Information) can be guaranteed if the sweat rate remains below 7.6 µL min^−1^ cm^−^^2^. It is worth noting that compensatory effects resulting from the occlusion of neighboring sweat glands increase the local sweat rate.^[^ ^5^ ^]^ The regional sweat rates considering the compensatory effects are determined to be up to 3 µL min^−1^ cm^−^^2^ at maximum exercise intensity, which confirms a negative lag time for real‐time detection.

Evaluation of the device efficacy under diverse levels of perspiration is carried out by introducing a 10 µL of 60 mm chloride solution into the device at flow rates ranging from 0.2 to 1.5 µL min^−1^ for mimicking sweat rate from 1.0 to 7.5 µL min^−1^ cm^−^^2^. Although lower flow rates (<0.3 µL min^−1^) cause reduced precipitation distances from evaporation and loss of space for subsequent sweat, the higher flow rates do not affect the analysis, as quantified by dye and precipitated distances (Figure 4c; Figure S6, Supporting Information). Because the glandular sweat flow rate ranges from 4 to 34 µg min^−1^ per gland,^[^ ^42^ ^]^ the device with an inlet size of 0.2 cm^2^ needs to cover the skin with a sweat gland density of at least 44 glands cm^−^^2^. Therefore, the commonly used body locations such as the head, forearm, abdomen, and back are also suitable for device placement. It should be noted that sealed devices attached to the skin could potentially cope with smaller sweat flow rates because the above results are from an open environment. Besides the range of (sweat) flow rate, sporadic sweating processes can also be handled by the developed device. In a simulated study, three droplets of chloride solution (5 µL each with a concentration of 75 mm) are dropwise added to the device, over a period from 5 to 30 min. The comparison in the precipitated distances between the one‐drop addition (DA) of 15 µL chloride solution and the series of three‐drop additions of 5 µL at different intervals of less than 10 min shows no significant statistical difference (Figure 4d). This results from minimized and negligible sweat evaporation from the channel, even during prolonged or intermittent episodes of sweating.

The bifluidic sweat chloride sensor also exhibits durable performance over time for up to 30 days and upon mechanical deformations. Despite slight discoloration in the B‐channel attributed to the oxidation of silver ions on the filter paper, accurate detection results of chloride concentrations are still obtained when compared with the freshly prepared devices during sports training (Figure S7a, Supporting Information). While the GOx in the glucose sensor is prone to inactivation, it can still maintain good activity when stored at low temperatures such as −20 °C for 3 days (Figure S7b, Supporting Information). To simulate the device's deformation worn in the complex human body curved surface, the bending of the device is introduced. With 3D‐printed molds with varying radii to simulate different bending conditions, the microfluidic device exhibits almost unchanged dye traveling front from added water of 15 µL even as the bending radius is reduced to 0.9 mm (Figure 4e). Although the device performance decreases as the bending radius is further reduced to 0.4 mm (fully bent), the distance of the dye traveling front could still recover after full bending and recovery (Figure S8, Supporting Information), indicating the high potential of the device for real‐world applications.

### 2.5 Feature Designs of the Bifluidic Sweat Device

**Figure 5:**
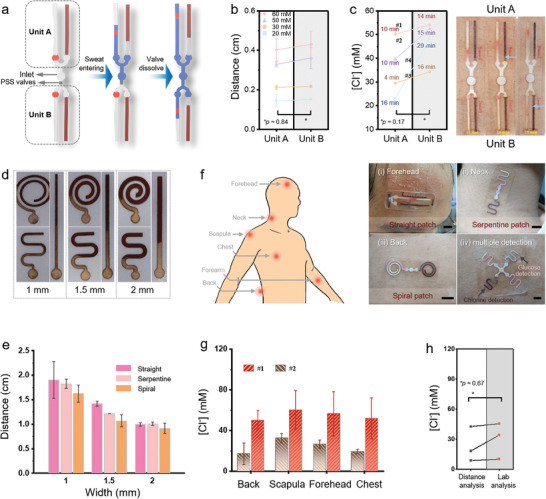
Feature designs of skin‐interfaced bifluidic sweat devices. a) Schematic showing the use of dissolvable polymer valves in wearable bifluidic devices for chrono‐sampling. b) Comparison in the chlorine detection between the devices without (Unit A) and with (Unit B) valves. c) Changes in the chloride concentration before and after exercise in four human subjects captured by the wearable bifluidic device with dissolvable polymer valves. d) Photograph of the various devices with different widths (1, 1.5, and 2 mm) and patterns for detecting chlorides (identical concentration in the same volume), and e) the resulting color change distance captured by these devices. f) Various shapes of devices mounted on different positions of the body: i) straight on the forehead, ii) single serpentine on the neck, iii) Archimedean spiral on the back, iv) cross with serpentine for multiple detections. Scale bar: 1 cm. g) Comparison in the chloride concentration on the back, scapula, forehead, and chest from two subjects. h) Comparison of the chloride concentration in sweat between the bifluidic sweat device and laboratory‐based analysis (with sweat collected from absorbing pads) (n = 3). *p < 0.05 from two‐tailed t‐test.

The combination of wearable bifluidic devices with soluble polymer valves could further provide sequential sampling and sweat biomarker analysis over time.^[^ ^43^ ^]^ The device is designed with two measurement units connected through a common inlet, with a 15% polystyrene sulfonate (PSS) dissolvable polymer valve in unit B whereas there is none in unit A. The time‐dependent detection in unit B is initiated after dissolving the polymer valve (**Figure** **5**a). Following the calibration of the modified device, the comparison in biomarker detection between two units indicates that the presence of the polymer valve does not have a significant impact on the measurements (Figure 5b). In the field testing with four subjects engaged in exercising, the sweat chloride concentration measured by the device mounted on the upper back increases after prolonged exercise due to decreased body water content (Figure 5c). The sweat chloride from the 10 min exercise is measured by unit A without a valve after 16 min, with the dye traveling front in unit A at the designated reading position (≈30 mm). At this point, sweat starts to enter unit B upon dissolving the polymer valve, followed by the detection of sweat chloride of ≈40 mm after 29 min. Increasing the polymer quantity could allow for an increased duration between the two measurements and incorporating additional sensing units yields more temporal measurement points.

The versatile manufacturing approach allows easy customization of paper‐fluidic channels with varying shapes and sizes in the resulting device platform. The narrower channels (1 mm in width) exhibit larger precipitation traveling distances (Figure 5d). With the same width, the channels changing from straight to serpentine and then to spiral patterns exhibit decreased precipitation traveling distance likely attributed to channel curvature (Figure 5e). The flexible thin devices with varying patterns can be easily mounted on different body locations with distinct advantages: straight devices on the forehead for easy readout and calibration, stretchable serpentine^[^ ^44^ ^]^ devices on the active‐moving areas such as the neck, and spiral devices on the back for increased collection volume (Figure 5f(i–iii)). The multi‐channel design also facilitates the simultaneous detection of multiple markers such as chloride and glucose (Figure 5f(iv)). The sweat [Cl^−^] on the upper backs of three subjects during exercise is lower than that at other locations (Figure 5g), which is attributable to higher reabsorptive capacity.^[^ ^45^ ^]^ The results are consistent with those from laboratory chloridometer measurements using an absorption pad (Figure 5h), confirming the accuracy of the device.

### 2.6 In Situ Biomarker Analysis of Sweat Samples

**Figure 6:**
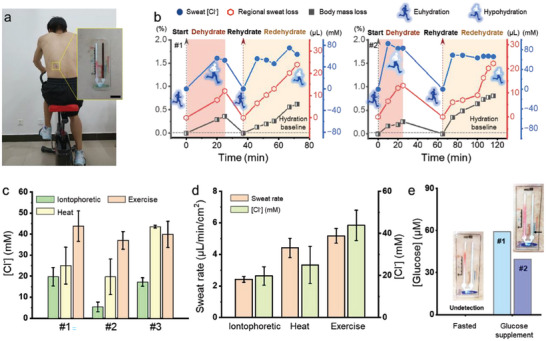
In situ biomarkers analysis from sweat samples of human subjects. a) Photograph of a healthy individual exercising on a stationary bike in a controlled environment. Scale bar: 1 cm. b) Sweat loss (or collected sweat), body weight loss, and sweat chloride concentration before and after rehydration of two subjects during indoor exercise. Comparison in the c) sweat chloride concentration and d) sweat rate from the chemical‐induced, exercise‐induced, and passive heat‐induced sweating. e) Comparison of the sweat glucose concentration before and after glucose supplementation from two subjects.

It is critical to maintain proper fluid and electrolyte balance for preserving physical and cognitive performance, particularly for professional athletes.^[^ ^46^, ^47^ ^]^ As sweat rates and electrolyte concentrations vary widely during training and competition, personalized fluid and electrolyte replenishment strategies based on individualized sweating profiles (e.g., sweat volume and loss of sodium [Na^+^]) are recommended. Monitoring chloride [Cl^−^] levels in sweat serve as a feasible alternative to tracking [Na^+^], as there is a significant correlation between the concentration of whole‐body sweat [Na^+^] and local sweat [Cl^−^].^[^ ^48^, ^49^ ^]^ For two subjects performing stationary bike exercises in a controlled environment (temperature of 25 °C and humidity of 67%), the local sweat [Cl^−^] and local sweat loss from the devices mounted on their waist (**Figure** **6**a) directly correlate with the body mass loss during dehydration, rehydration, and redehydration (Figure 6b). After 30 min of exercise, both sweat loss and [Cl^−^] increase with body mass loss, resulting in a state of mild dehydration. The participants subsequently rehydrated properly by consuming water equivalent to their total body mass loss, with the hydration baseline returned. Using a new device at the original skin site further captures fluid losses of 0.6% and 0.8% body mass after exercising for 35 and 60 min (without rehydration) for Subjects 1 and 2, respectively. During this redehydration process, the sweat [Cl^−^] levels remain high at 60–80 mm. The increased serum [Cl^−^] levels stimulate chloride ion transport into the sweat gland during hypohydration. Although the device cannot directly measure the absolute hydration level, the customizable replenishment water reminders can be tailored for each individual based on the change in hydration level measured by the device during dehydration.

Compared to exercise‐induced sweating, pharmacologically induced sweating (e.g., through pilocarpine iontophoresis) or passive heat‐induced sweating provides higher precise and presents less physical taxing on patients in routine testing and medical diagnostics. The sweat chloride concentration induced by pilocarpine iontophoresis is significantly lower compared to that from the other two (Figure 6c), which is consistent with previous reports (8–47.6 mm from pilocarpine iontophoresis^[^ ^28^ ^]^ versus 21–81 mm from exercise‐induced sweat^[^ ^50^ ^]^ in healthy human). The difference in chloride concentration between these methods can be attributed to local cholinergic stimulation of sweat glands from pilocarpine iontophoresis, whereas sweating induced by exercise or heat stress involves other mediators both locally and centrally.^[^ ^51^ ^]^ Moreover, the increased activity of the sympathetic nervous system leads to enhanced activity of basolateral Na^+^‐K^+^−2Cl^−^ co‐transporters and increased sweat chloride concentration.^[^ ^52^ ^]^ Increased concentrations of sweat electrolytes also seem to correlate with higher sweat rates across the three methods (Figure 6d). It is widely accepted that chlorine is reabsorbed by sweat glands as sweat passes through sweat ducts, but the capacity of sweat glands to absorb chlorine is relatively low in cases of high sweat rate, leading to increased sweat chloride concentration as the sweat rate increases.^[^ ^53^, ^54^ ^]^

Glucose diffusion from the blood to sweat involves the traversal of capillaries, interstitial fluid, and sweat glands.^[^ ^9^ ^]^ As a result, the glucose concentration in sweat is ≈1–2% of that in blood,^[^ ^13^ ^]^ but the close correlation between the two supports the use of sweat glucose for non‐invasive measurements. To this end, the designed bifluidic glucose device is demonstrated to measure sweat glucose concentration following glucose supplementation. Specifically, subjects fasting for 8 h prior to receiving a glucose supplement exhibited very low levels of sweat glucose that fell below the device's detection limit, as evidenced by the absence of precipitate color in the B‐Channel. After drinking a 60 g glucose rehydration solution over the course of an hour, a clear blue precipitate formed in the B‐Channel indicating an increased sweat glucose concentration to ≈35–60 µm (Figure 6e). The measured results are consistent with the previously reported results: 5–10 µm from fasting and 20–50 µm following glucose supplement.^[^ ^55^ ^]^

## 3 Discussion

In summary, the skin‐interfaced bifluidic sweat device can achieve real‐time sampling of sweat and quantitative visualization of biomarkers. With the sweat rate and volume measured from one channel in the paper‐based microfluidics, the colorimetric detection of the biomarker in the other channel provides the direct readout of the concentration, regardless of sweat gland secretory pressure. The design without an outlet minimizes external contamination and sample evaporation, increasing accuracy and rendering its use in extreme cases such as aquatic or sports environments without backpressure effects. The low‐cost and scalable fabrication method also allows customization of the device for different users (e.g., infant or adult) on various body positions.

The distance‐based colorimetric detection combined with capillary action through cellulose fibers provides a rapid and intuitive distance/length reading to detect biomarker concentrations. A wide range of biomarkers can be detected with pre‐deposited dry reagents to produce colored bands through enzymatic reactions (e.g., lactate and alcohol), ionophore reactions found in nanospheres (e.g., potassium and calcium), precipitation (e.g., chloride), or metal complexes (e.g., iron and copper). Although some needed enzymes (e.g., GOx) have low sensitivity and limited storage performance in the ambient environment, improved activity and stability can be possible with recently reported nanozymes due to increased specific surface areas.

The irreversible nature of distance‐based colorimetric detection makes it difficult to use wearable colorimetric sensors for long‐term monitoring of sweat analytes over days or weeks. A potential solution is to explore multiple reaction chambers connected by the microfluidic channel and controlled by passive capillary bursting valves or soluble polymer valves to separate measurements in time for successive colorimetric analysis. It is also possible to use heating powder, fluorescent powder, or chemesthetic irritants to remind subjects of replacement upon sweat saturation in the device. Nonetheless, the demonstrated sensing principle and device platform provide promising opportunities for simple yet accurate, continuous measurements of various biomarkers in sweat and other biological fluids for health monitoring and clinical disease diagnosis.

## 4 Experimental Section

### Materials

Whatman filter paper No. 1 was obtained from Whatman International Ltd. (Maidstone, England). Silver nitrate (>99.8%) was obtained from Tongbai Xinhong Silver Products Co., Ltd. (China). Sodium chloride (>99.5%) was obtained from Tianjin Kermel Chemical Reagent Co., Ltd. (China). Potassium chromate (>99%) was obtained from Sinopharm Chemical Reagent Co., Ltd. (China). Iron(III) chloride (>98%), glucose, ascorbic acid (>99.99%), potassium ferrocyanide trihydrate (>98%), and glucose oxidase from Aspergillus niger (>180 U mg^−1^) were obtained from Aladdin Industrial Corporation.

### Preparation of Paper‐Based Microfluidic Devices

The fabrication of the bifluidic sweat device started with homogeneous deposition of colorimetric detection reagents along the B‐channel using an inkjet printer (Canon IX6880) with the reagent loaded in its refillable cartridge (Figure S1, Supporting Information). Next, the filter paper was laser‐machined into a bi‐channel pattern using a laser engraver (JK‐DK40, Wenhao Co., Ltd., China). Quantitative pipetting of 0.7 µL dye/enzyme into the circular loading areas (4 mm in diameter) of different channels was followed by drying at room temperature for 5 min. The PET stickers with printed scale marks and paper‐based microfluidic layers were assembled on a double‐sided adhesive tape through stencil lamination, and then encapsulated with a 100 µm‐thick PET film. The assembled device was laser‐machined into a rectangular shape (W × L of 20 mm × 53 mm) with rounded corners (R of 5 mm) to complete the device preparation.

Silver chromate was used as the colorimetric reagent to detect chloride. The silver chromate precipitate was inkjet generated by printing 50 mm potassium chromate and 250 mm silver nitrate on the filter paper for 20 cycles. After each printing cycle, the paper surface was exposed to a stream of hot air from a hair dryer (power of 1800 W) before the next printing cycle. Next, the filter paper with printed reagent was laser‐machined into the bi‐channel pattern (1.5 mm wide) using a laser engraver, with the brown silver chromate precipitate just in the B‐channel. The colorimetric reagent of PW to detect glucose was prepared in a similar manner. The 5 mm ferric chloride and the 5 mm potassium ferrocyanide were printed on the filter paper for 10 cycles each. The reaction of these two formed a PB precipitate. Printing the 100 mm ascorbic acid with 10 cycles at the same position further reacted with the PB to form PW. Laser‐machining the printed filter paper into a bi‐channel pattern (1 mm wide) was followed by washing with distilled water and drying at room temperature for 1 h. Dropwise adding 0.7 µL of 500 U mL^−1^ GOx solution to the sample area and drying at room temperature for 10 min completed the preparation.

### Characterization and Measurements

SEM images were obtained with an ultrahigh‐resolution field‐emission SEM (SU5000, Hitachi, Japan). A smartphone (OPPO K9x) was used for taking images and recording videos. ImageJ software was used to quantify the flowed distance of the liquid, dye, and precipitate color (in the V‐channel or B‐channel). The measurements were repeated in at least four replicates to characterize the device's performance.

### Experiments on Human Subjects

All experiments on human subjects were conducted under the approval of the Institutional Review Board at The Pennsylvania State University (protocol number STUDY00020880). Informed consent was obtained from all human subjects. The skin of the human subjects was first washed with soap and running tap water and dried with disposable paper. The stationary bike exercise was performed in a controlled environment (temperature of 25 °C and humidity of 67%) with the device mounted on the waist of the two subjects. Before the euhydration experiment, the subjects were asked to drink 500 mL of water and rest for about half an hour. The sweat chloride concentration and volume over time were captured by the developed device, whereas the body weight loss was measured by a body weight scale with 1‐g precision. The experiment was ended after the collected sweat reached 12 µL. The subject recovered to their initial body weight after consuming the supplemented water. After resting (12 min for subject #1, 40 min for subject #2), a new device from the same back of fabrication was used in the next cycle of the experiment with exercise‐induced sweat. To compare the three different sweat‐inducing methods, the sweat rate and chloride concentration from three subjects were measured, with the sweat induced by three different methods. All experiments were conducted under euhydration. The exercise‐induced sweating was carried out outdoors and the passive heat‐induced sweating was carried out in the indoor sauna, with the device placed on the back of the subject for both cases. The chemical‐induced sweating was carried out with pilocarpine iontophoresis near the device on the forearm by following the previously reported methods.^[^ ^56^ ^]^ In brief, the prepared pilocarpine nitrate alkaline hydrogel and sodium nitrate hydrogel on two copper foil electrodes served as the positive and negative electrodes, respectively. Applying a current of 0.4 mA for 5 min from a direct‐current power supply over the two electrodes yielded continuous sweating of ≈30 min. The data and time were recorded after 10 µL of sweat was collected by the device.

## Conflict of Interest

The authors declare no conflict of interest.

## Author Contributions

M.D. and X.L. contributed equally to this work. X.W. and M.D. conceived the idea and designed the experiments. M.D. designed and optimized the paper‐based device. M.D., X.L., K.S., H.Y., and W.W. contributed to devices’ fabrication and characterization. M.D., K.S., X.D., and X.W. contributed to the data analysis and interpretation. X.W., M.D., and H.C. wrote the paper, and all authors provided feedback. X.W., X.O., and H.C. supervised the study.

## Supporting information

Supporting Information

## Data Availability

The data that support the findings of this study are available in the supplementary material of this article.
