# Supplementary material for: Skin‐Interfaced Bifluidic Paper‐Based Device for Quantitative Sweat Analysis
Source: Adv Sci (Weinh). 2023 Dec 22;11(10):2306023. doi: 10.1002/advs.202306023 (PMC10933605; doi:10.1002/advs.202306023)
Supplement: Supplementary file 1 — Supporting Information [file ADVS-11-2306023-s001.pdf]

ADVANCED SCIENCE
Open Access

# Supporting Information

for *Adv. Sci.*, DOI 10.1002/advs.202306023

Skin-Interfaced Bifluidic Paper-Based Device for Quantitative Sweat Analysis

*Muhan Deng, Xiaofeng Li, Kui Song, Hanlin Yang, Wenkui Wei, Xiaojun Duan, Xiaoping Ouyang, Huanyu Cheng* and Xiufeng Wang**

# Supplementary Information

## Skin-interfaced bifluidic paper-based device for quantitative sweat analysis

Muhan Deng[1]†, Xiaofeng Li[1]†, Kui Song[2], Hanlin Yang[1], Wenkui Wei[1], Xiaojun Duan[4], Xiaoping Ouyang[1], Huanyu Cheng[3*], Xiufeng Wang[1*]

[1] School of Materials Science and Engineering, Xiangtan University, Xiangtan, Hunan 411105, China

[2] Department of Engineering Science and Mechanics, Xiangtan University, Xiangtan, Hunan 411105, China

[3] Department of Engineering Science and Mechanics, The Pennsylvania State University, University Park, Pennsylvania 16802, United States

[4] Hunan Provincial Children's Hospital, Changsha, Hunan 410000, China

[*]To whom any correspondence should be addressed: Huanyu.Cheng@psu.edu (H.C.) or onexf@xtu.edu.cn (X.W.).

†These authors contributed equally to this work.

# Table of Contents

**Supplementary Notes** .......... **3**

Supplementary Note 1. Kinetics of sweat flow in the paper-based microchannel. .......... 3

**Supplementary Figures** .......... **5**

Supplementary Figure 1. Schematic showing the fabrication procedure of the bifluidic device. .......... 5

Supplementary Figure 2. Optical images of the skin-interfaced bifluidic device .......... 6

Supplementary Figure 3. Flow characteristic of paper-based microfluidic device. .......... 7

Supplementary Figure 4. The measured red dye traveling distance as a function of the solution with different volumes (5, 7, 10, 12, 15, and 20 μL) and varying chloride concentrations (10, 20, 30, 50, 60, 75, and 100 mM). .......... 8

Supplementary Figure 5. Optical images of the device attached to the upper back of a human subject exercising for (i) 20, (ii) 23, and (iii) 29 min. .......... 9

Supplementary Figure 6. Optical images of the dye and precipitated distances, for 10 μl solution with 60 mM chloride injected at various flow rates: 0.2, 0.3, 0.4, 0.5, 0.8, 1.0, and 1.5 μL/min. .......... 10

Supplementary Figure 7. Long-term stability of (a) the chloride sensor and (b) the glucose sensor. .......... 11

Supplementary Figure 8. Comparison in the dye traveling front in the device before and after bending for 1, 2, and 3 times. .......... 12

**References** .......... **13**

## Supplementary Notes

### Supplementary Note 1. Kinetics of sweat flow in the paper-based microchannel.

The capillary force per unit area in the porous media (e.g., paper) can be determined using the Young-Laplace equation[1,2]

$$P_{capillary} = P_c = \frac{2\gamma\cos\theta}{r}, \quad \text{(S1)}$$

where $\gamma$ is the surface tension coefficient, $\theta$ is the contact angle, and $r$ is the equivalent radius of the porous medium. The saturated liquid flowing inside the paper channel can be modeled as a viscous flow described by Darcy's law[3], with its viscous flow resistance expressed as

$$\Delta P_D = \frac{\rho\mu}{K} v l_{\mathrm{liq}} = \frac{\rho\mu}{K} l_{\mathrm{liq}} \frac{\mathrm{d}l_{\mathrm{liq}}}{\mathrm{d}t}, \quad \text{(S2)}$$

where $\mu$ is the kinetic viscosity of the liquid, $\rho$ and $K$ are the intrinsic porosity and permeability, $v$ is the average flow velocity, and $l$ is the imbibition length.

The process of liquid imbibition in porous media is regulated by the equilibrium between the capillary force and the viscous force, leading to:

$$\frac{\rho\mu}{K} l_{\mathrm{liq}} \frac{\mathrm{d}l_{\mathrm{liq}}}{\mathrm{d}t} = \frac{2\gamma\cos\theta}{r}. \quad \text{(S3)}$$

According to the solution of the Poiseuille flow[4], the pressure difference in the circular tube with a laminar flow can be obtained as $\Delta P = \frac{8\mu l_{\mathrm{liq}}}{\pi r^4} Q = \frac{8\mu l_{\mathrm{liq}}}{r^2} v$. Comparing with Eq. (S2) gives $r = \sqrt{\frac{8K}{\rho}}$. With an imbibition length of zero at $t = 0$, the solution to Eq. (S3) can be obtained as the Lucas-Washburn equation[5]

$$l_{\mathrm{liq}} = \sqrt{\frac{4\gamma K\cos\theta}{\rho\mu r}} t^{1/2} = \sqrt{\frac{\gamma r\cos\theta}{2\mu}} t^{1/2}. \quad \text{(S4a)}$$

Considering a consistent lag between the advancing fronts of the dye ($l_{\mathrm{dye}}$) and the liquid ($l_{\mathrm{liq}}$), a retardation factor $R_{\mathrm{f}}$ can be used to correlate the two for a given device[6,7]: $R_{\mathrm{f}} = \frac{l_{\mathrm{dye}}}{l_{\mathrm{liq}}}$. Hence, the kinetic behavior of the dye wicking front can be expressed as

$$l_{\mathrm{dye}} = R_{\mathrm{f}}\sqrt{\frac{\gamma r \cos\theta}{2\mu}}t^{1/2} = kt^{1/2}. \tag{S4b}$$

The flow rate of the liquid in the channel with a height of $\delta$ and a width of $b$ can be given as

$$Q_i = \delta b v \rho = \delta b \rho \frac{\mathrm{d}l}{\mathrm{d}t} = \delta b\sqrt{\frac{\gamma \rho K \cos\theta}{\mu r}}t^{-1/2} \tag{S5a}$$

and the total volume of the collected liquid in the channel is

$$V_i = \int Q_i \mathrm{d}t = 2\delta b\sqrt{\frac{\gamma \rho K \cos\theta}{\mu r}}t^{1/2}. \tag{S5b}$$

Assuming a constant regional sweat rate per unit area of $\eta$ on the human body, the flow rate from sweating over the device inlet with an area of $A$ can be denoted as

$$Q_s = A\eta. \tag{S6a}$$

The sweat volume collected over time is then obtained as

$$V_s = \int Q_s \mathrm{d}t = A\int \eta \mathrm{d}t. \tag{S6b}$$

When the flow rate from sweating is higher than the sweat flow rate in the microfluidics, a portion of the secreted sweat is not wicked by the device for an extended duration, causing a delay in sweat sampling with a lag time of $\Delta t = t_2' - t_2$ (Fig. 4b).

## Supplementary Figures

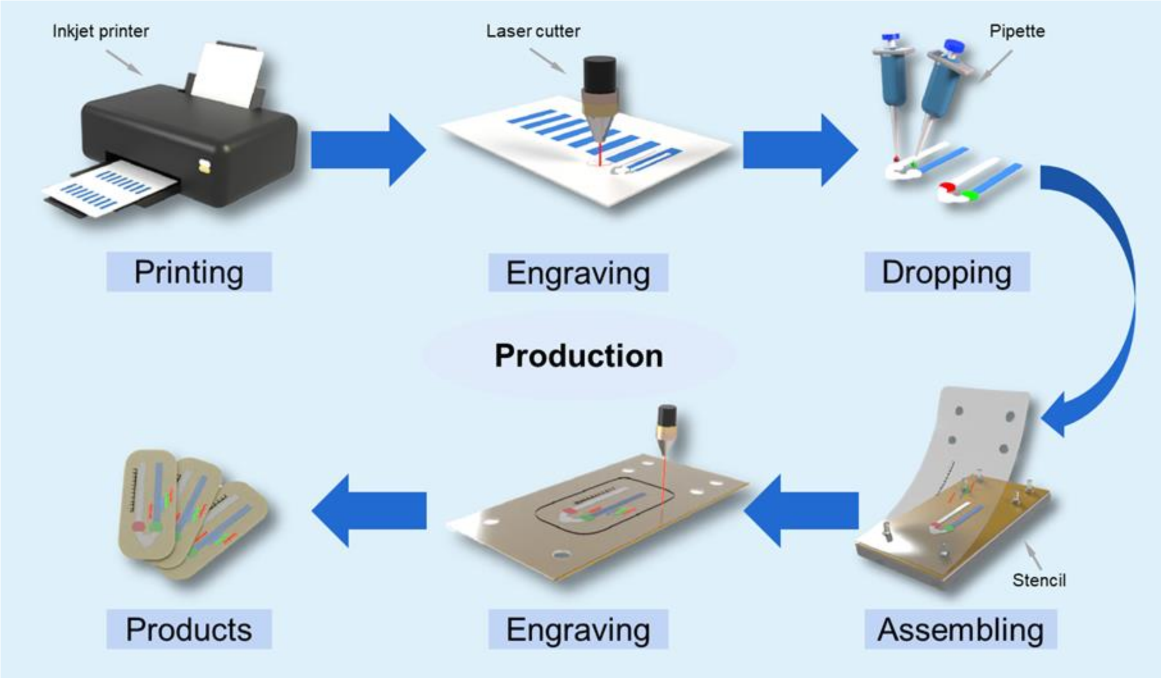


**Supplementary Figure 1. Schematic showing the fabrication procedure of the bifluidic device.**

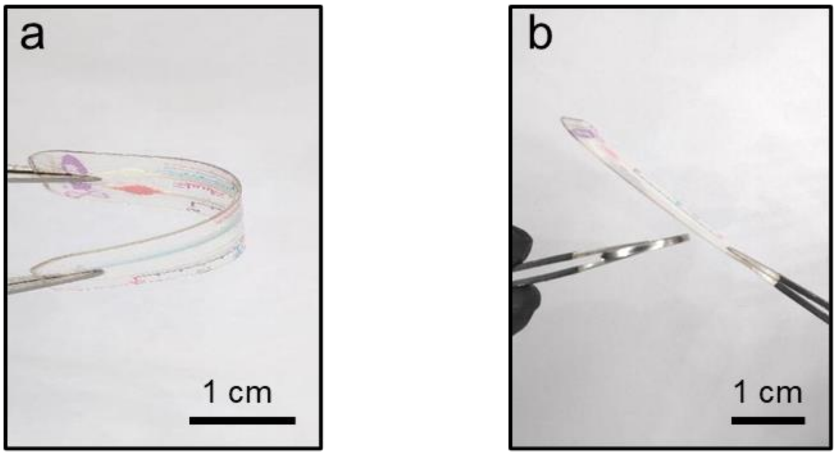


**Supplementary Figure 2. Optical images of the skin-interfaced bifluidic device. (a)** after bending and **(b)** its thickness comparison with a Chinese coin of one dime.

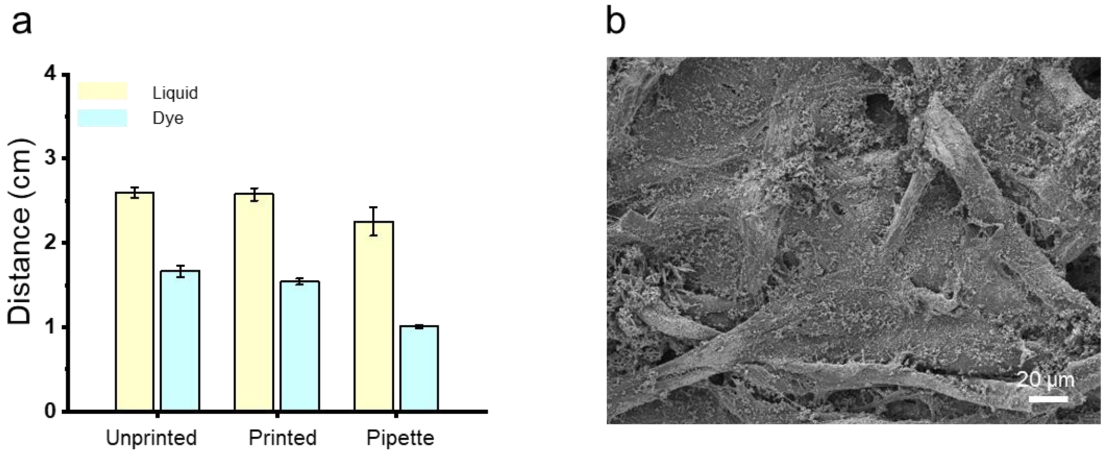


**Supplementary Figure 3. Flow characteristic of paper-based microfluidic device. (a)** Comparison of the flow liquid and dye traveling distance between pristine, inkjet-printed, and pipette-dropped filter papers. **(b)** SEM image of the pipette-dropped filter paper.

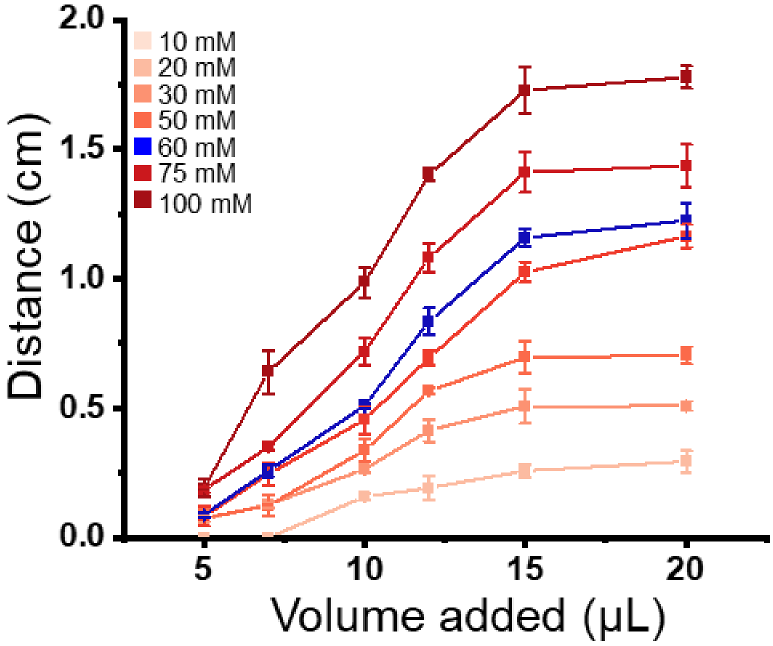


**Supplementary Figure 4. The measured red dye traveling distance as a function of the solution with different volumes (5, 7, 10, 12, 15, and 20 μL) and varying chloride concentrations (10, 20, 30, 50, 60, 75, and 100 mM).**

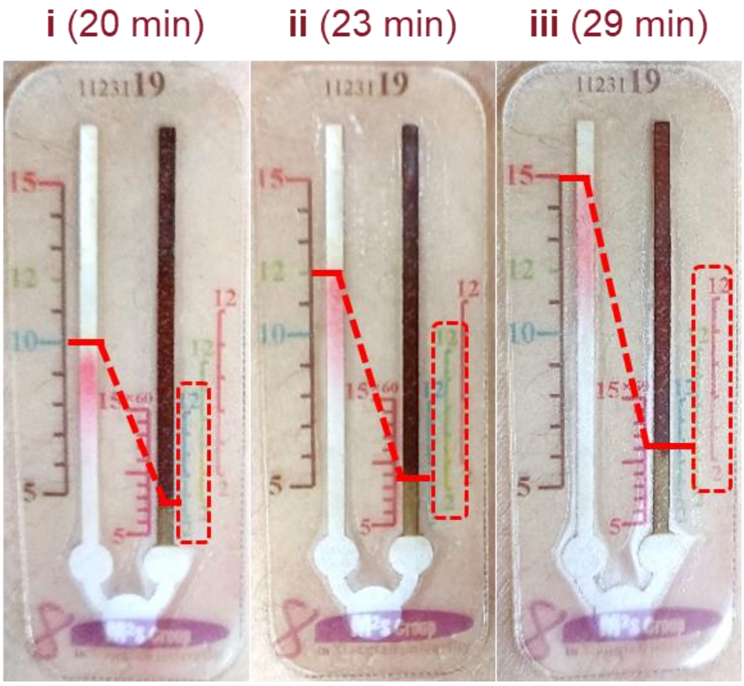


**Supplementary Figure 5. Optical images of the device attached to the upper back of a human subject exercising for (i) 20, (ii) 23, and (iii) 29 min.**

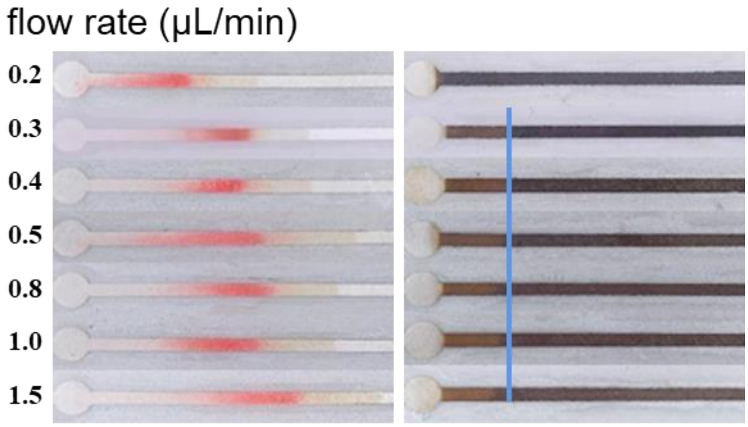


**Supplementary Figure 6. Optical images of the dye and precipitated distances, for 10 µl solution with 60 mM chloride injected at various flow rates: 0.2, 0.3, 0.4, 0.5, 0.8, 1.0, and 1.5 µL/min.**

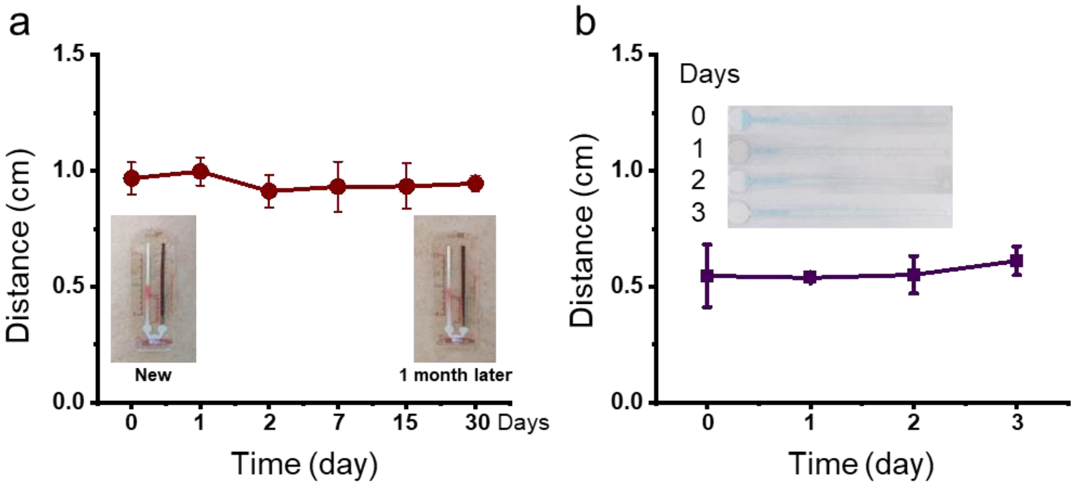


**Supplementary Figure 7. Long-term stability of (a) the chloride sensor and (b) the glucose sensor.**

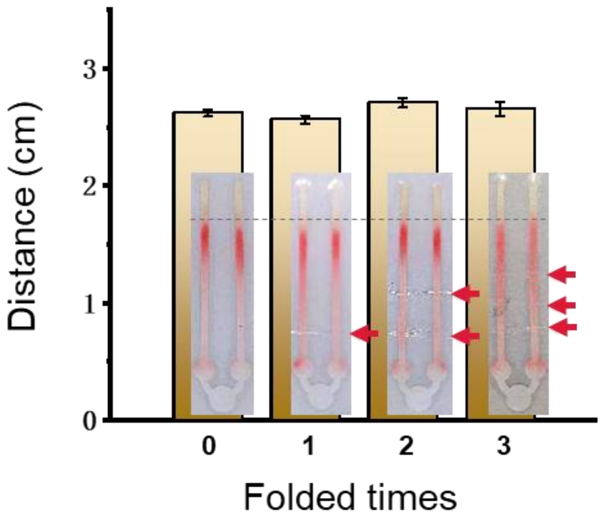


**Supplementary Figure 8. Comparison in the dye traveling front in the device before and after bending for 1, 2, and 3 times.**

## References


1. Laplace, P. S. *Traité de mécanique céleste vol. 5* (Chez JBM Duprat, Augustins, 1825.).
2. Young, T. An essay on the cohesion of fluids. *Philos. Trans. R. Soc. London* **95**, 65-87 (1805).
3. Darcy, H. *Les fontaines publiques de la ville de Dijon: exposition et application des principes à suivre et des formules à employer dans les questions de distribution d'eau. Vol. 1* (Victor Dalmon, 1856).
4. Davey, A. & Drazin, P. The stability of Poiseuille flow in a pipe. *J. Fluid Mech.* **36**, 209-218 (1969).
5. Washburn, E. W. The dynamics of capillary flow. *Phys. Rev.* **17**, 273 (1921).
6. Jaitpal, S., Naik, P., Chakraborty, S., Banerjee, S. & Paul, D. Exploring the concentration-dependent transport and the loss of rhodamine B, tartrazine, methylene blue, and amaranth dyes in common paperfluidic substrates. *Result. Surf. Interface.* **6**, 100034 (2022).
7. Wendenburg, S., Nachbar, M.-L. & Biesalski, M. Tailoring the retention of charged model compounds in polymer functionalized paper-based microfluidic devices. *Macromol. Chem. .Phys.* **218**, 1600408 (2017).
